# On the wings of dragons: Wing morphometric differences in the sexually dichromatic common whitetail skimmer dragonfly, *Plathemis lydia* (Odonata: Libellulidae)

**DOI:** 10.1371/journal.pone.0303690

**Published:** 2024-05-29

**Authors:** Andrew O. Rubio, Ashley M. Dye, Kyle E. Ifill, Kyle Summers

**Affiliations:** 1 Department of Biology, East Carolina University, Greenville, North Carolina, United States of America; 2 Department of Biology and Biochemistry, University of Houston, Houston, Texas, United States of America; Laboratoire de Biologie du Développement de Villefranche-sur-Mer, FRANCE

## Abstract

Sexual dimorphism is common throughout the animal kingdom, leading to sex-specific phenotypic differences. The common whitetail skimmer dragonfly, *Plathemis lydia* (Drury, 1773), is sexually dichromatic, where males of this species display a conspicuous white abdomen and females display a dark brown abdomen. Differences in abdomen conspicuousness between male and female *P*. *lydia* are likely attributed to differences in selective pressure where males use their white conspicuous abdomen during male-male territorial chases. We hypothesized that male *P*. *lydia* would exhibit wing morphology adaptations to better offset the costs of predation and territoriality and that these adaptations would differ from females. We used field-collected images to quantify differences in body length, wing length, wing area, wing shape, and wing loading between male and female *P*. *lydia*. Our results show that male *P*. *lydia* have significantly shorter fore and hind wings relative to body size with a higher wing loading when compared to females. We also found that male *P*. *lydia* have narrower and pointier fore and hind wings compared to females. These results are consistent with the idea that males are adapted for faster flight, specifically higher acceleration capacity, and higher agility whereas females are adapted for higher maneuverability.

## Introduction

Evolutionary biologists have long been captivated by the variation in coloration seen across the animal kingdom and research on coloration has significantly advanced our understanding of evolutionary processes. Inter- and intraspecific signaling are pervasive and have evolved as central elements of key ecological and evolutionary processes. For instance, signaling is essential for species recognition, mate choice, and predator avoidance [1–3]. Intraspecific color divergence has been a central focus of evolutionary research [4–6]. Sexual dichromatism, a form of sexual dimorphism, may occur when selection favors males with exaggerated signals, such as brighter and more colorful morphological traits for mating and male-male competition, whereas selection on females favors dull coloration for background matching [7–12].

Conspicuous communication signals, such as bright and intense coloration, can impose significant costs as they are often used by visual predators to identify prey [13–15]. Therefore it is axiomatic that, compared to females, the risk of detection and predation are high for conspicuous males [16–20]. Nonetheless, evidence suggests that certain adaptations, such as attack deflection, altered behavior, signal partitioning, and communicating privately (i.e. in a manner that is less conspicuous to predators than to conspecifics) can offset the negative consequences of exhibiting vivid signals [14, 21–25]. For instance, Chotard et al. [25] found that the conspicuous hind wing wingtails in the scarce swallowtail butterfly function to deflect a predator’s attack away from vital body parts. Veins in these regions were found to be less resistant to tensile force and break sooner while maintaining fore wing integrity and allowing for an escape. Such adaptations are favored by natural selection as they permit the survival of conspicuous prey [26]. Other processes besides predation risk may also drive the evolution of adaptive morphological traits. For instance, organisms that exhibit sexual selection may undergo adaptation to offset pressures from male-male competition and female mate choice. Male victors from competitions with male rivals benefit by gaining increased mating opportunities [27]; hence, morphological features vital for male-male competition are likely under positive selection pressure in the context of sexual selection.

Wing shape can be an excellent indicator of adaptation to selective pressures such as predator avoidance [25], sexual selection [28], migration [29, 30], and foraging strategies [31]. For instance, wings that are long and narrow are associated with increased speed and agility (the speed at which a turn can be made), whereas wing shapes that are short and wide are associated with reduced speed and increased maneuverability (the radius of the turn that can be made) [32–39]. Specifically, in terms of increased speed, wings that are long and narrow are associated with high acceleration capacity [40]. In addition, body and wing sizes greatly impact the flight performance of flying organisms through changes in wing loading [41]. Wing loading is the measurement of the amount of weight carried by each wing in flight [42].

The order Odonata, composed of dragonflies and damselflies, is one of the oldest lineages of winged insects known for their large size, vivid coloration, conspicuous diurnal behavior, and flight performance [43]. The morphology and shape of dragonfly wings are highly variable [35]; possibly indicating divergent selective pressures on wing shape. The flight performance of a dragonfly can be substantially affected by variation in the shape of fore and hind wings. Johansson et al. [37] found that migratory populations of the globe skimmer dragonfly (*Pantala flavescens*) had a broader wing base and an overall more slender wing shape compared to non-migratory populations, suggesting that wing shape is important for successful long distance migration. As for wing loading, dragonflies with low wing area relative to body mass exhibit high wing loading which increases flight speed and organisms with high wing area relative to body mass exhibit low loading which increases maneuverability [41]. Organisms with high wing loading exhibit turns with large radius, whereas, organisms with low wing loading exhibit turns with small radius [42].

**Fig 1:**
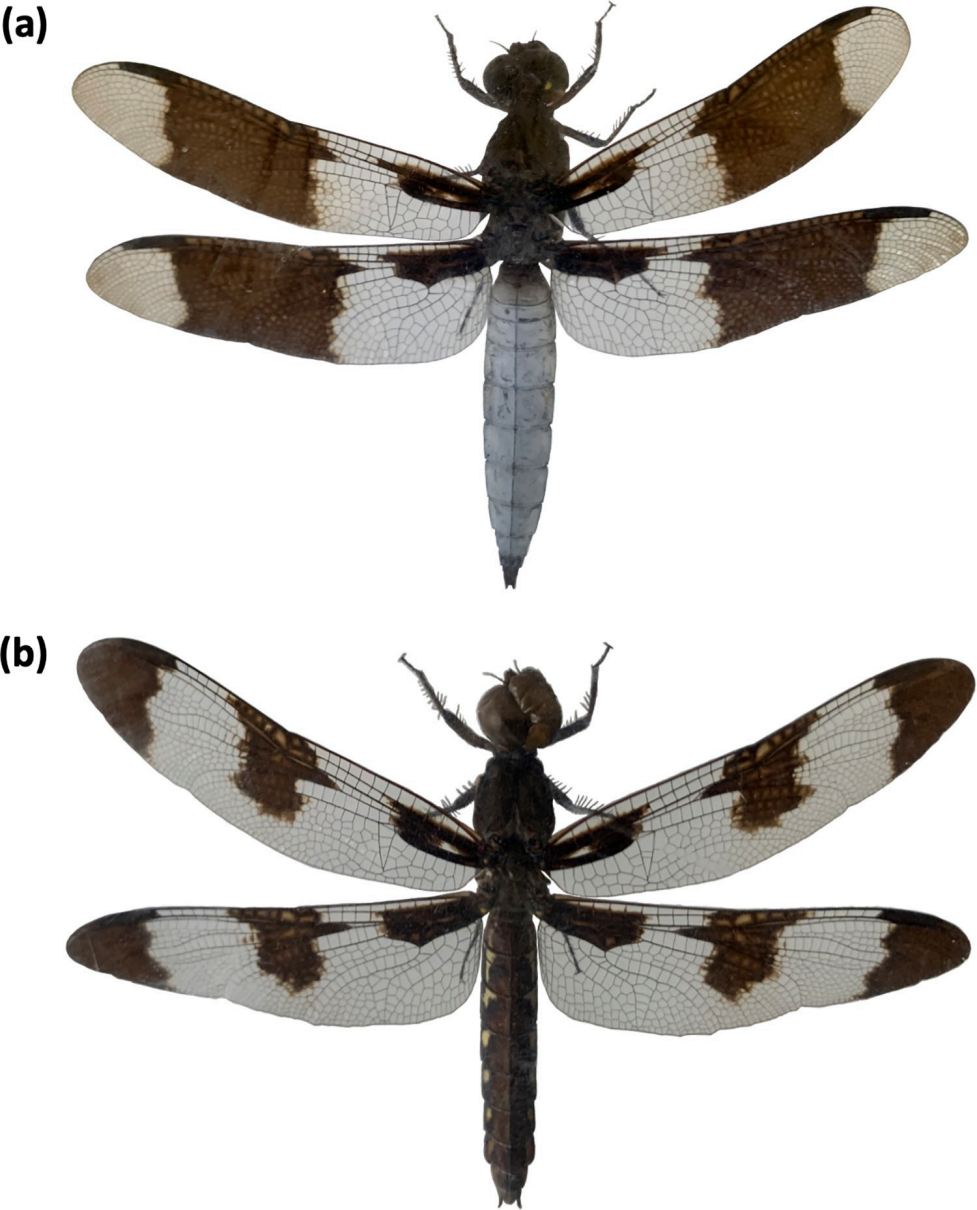
Dorsal view of a **(a)** male common whitetail skimmer dragonfly and **(b)** female common whitetail skimmer dragonfly (*Plathemis lydia*).

The common whitetail skimmer dragonfly, *Plathemis lydia* (Drury, 1773), is sexually dichromatic; males exhibit a conspicuous white abdomen with a median dark band on translucent fore and hind wings, whereas females exhibit a dark brown abdomen with median and terminal dark spots on translucent fore and hind wings (Fig 1). Differences in abdomen and wing coloration between male and female *P*. *lydia* are likely attributed to differences in selective pressure. Experiments on clay models suggest that white coloration is more conspicuous to avian predators than duller and darker coloration. For instance, Marshall et al. [44] found that white painted lizard clay models were detected and attacked more often than brown and gray painted lizard clay models. Another study found that the luminance contrast and attack rate were higher on lizard clay models with white markings when compared to lizard clay models with purple markings [45].

Male *P*. *lydia* use their white conspicuous abdomen during male-male territorial chases, however, they do not use them during courtship with females [46, 47]. Considering that the visibility of male *P*. *lydia* is enhanced, it is likely that they are detected more frequently by predators than females. Predator evasion and male-male territory chases presumably pose the same selective pressures on wing size and shape. For instance, increased acceleration, speed, and agility are essential flying abilities for evading predation [48]. Such flying abilities may allow male *P*. *lydia* to accelerate to a maximum speed quickly and evade predation despite increased detection. As for male-male territorial chases, increased speed is associated with effectively herding rival males out of claimed territories [47, 49]; hence, it is likely that increased speed is an essential flying ability for male *P*. *lydia*. Considering that females are not under the same pressures as males, it is likely that female *P*. *lydia* favor wing shapes that increase maneuverability and gliding instead of speed and agility. Therefore, we hypothesized that male dragonflies will exhibit wing adaptations associated with increased acceleration, speed, and agility to escape from frequent predator attacks and perform male territorial chases, whereas females will exhibit wing adaptations associated with increased maneuverability. We predicted that wing male *P*. *lydia* will exhibit narrower and pointier fore and hind wings and higher wing loading compared to females.

## Methods

### Specimen collection

Male and female *Plathemis lydia* were caught with insect nets during the summer of 2022 in Greenville, North Carolina, United States of America (35.6069° N, 77.3665° W). We placed each individual in a clear plastic bag with a reference scale on top and photographed with an iPhone X_R_. We immediately released all captured specimens once photographs were taken. We examined dragonfly wing venations from each photograph to prevent measuring recaptured dragonflies. No permits or voucher specimens were required as this study was carried out on public lands and did not involve collection or preservation.

### Body length, wing length, wing area, and wing loading

We used the program ImageJ version 1.53 [50] to estimate the length and area of the fore and hind wings of each photographed *P*. *lydia* dragonfly. Body length was measured from the tip of the head to the end of the abdomen (excluding appendages). We used the distance between landmarks 0 and 5 to estimate the length of the fore and hind wing (S1A Fig). To measure the area of the fore and hind wings, we used imageJ to trace the outline of the wing shape. These points were carefully taken from the interior side of the most exterior venations (S1B Fig). In addition, a straight line connected gaps on the proximal side of the wings (landmark 1 and 10 on fore wings; landmark 1 and 11 on hind wings). We measured the body size and wing length of 36 individuals (18 males and 18 females). Due to wing damage, we were not able to calculate wing area and wing loading for all 36 samples. Instead, we calculated wing area and wing loading for 32 individuals, 18 males and 14 females. In addition, due to the dark banding on the fore and hind wings, we analyzed the shape of 9 fore and 10 hind wings (no duplicate wings for any individuals).

We used a Shapiro-Wilk [51] test to determine if the residuals for body length, fore and hind wing length, and fore and hind wing area were normally distributed. We calculated relative fore and hind wing length by dividing mean fore and hind wing length by body length and calculated relative fore and hind wing area by dividing mean fore and hind wing area by body length. Wing loading is defined as body mass relative to wing length as an indicator of flight efficiency and maneuverability [52]. While we were unable to collect the dry body mass, we used body length^3^ as an estimate of body weight and hence a replacement of body mass to calculate total wing loading. To calculate total wing loading, we divided the estimated body weight by the sum of the area of all four wings for each individual $(\frac{estimated\;weight\;(body\;length^{3})}{fore\;and\;hind\;wing\;area}).$

We performed a Welch’s t-test to evaluate whether there was a significant difference in body length between male and female *P*. *lydia* dragonflies. We used an analysis of covariance (ANCOVA) to test for differences in wing traits between male and female *P*. *lydia* while using body length as a covariate to control for body size. Specifically, we built models that fit log (fore and hind wing length), log (fore and hind wing area), and log (wing loading) as a function of log (body length), sex and their interaction. We performed all analyses above in R version 4.3.2 “Eye Holes” [53].

### Wing shape: Size variation and allometric effect

We used the R package StereoMorph [54] to obtain 10 landmark points on the fore wing and 11 landmark points on the hind wing from each photographed *P*. *lydia* dragonfly (S2 Fig). Depending on the sex, *P*. *lydia* dragonflies have median dark band or median and terminal dark spots on translucent fore and hind wings, and therefore, we used the program Darkroom version 6.3.2 to alter photo exposure (black, midtones, and white) in order to identify wing venations and collect wing landmark for shape analyses. We then used the program MorphoJ [55] to perform all wing shape analyses with collected landmarks. In order to directly compare fore and hind wing shape without the effect of size, we performed a generalized Procrustes analysis (GPA). A Procrustes analysis implements isomorphic scaling, translation, and rotation to determine the optimal fit for two or more landscaped shapes.

We then performed a Principal Component Analysis (PCA). We corrected for wing size by extracting residuals from a regression of procrustes distance to log centroid size before testing for shape difference between the fore and hind wings of male and female *P*. *lydia*. The use of residuals, to account for allometry, assumes a common allometric relationship in both sexes. Wing centroid size is the measure of size, calculated from the square root of the sum of squared distances from the centroid of all landmarks. Correcting for the effect of size on shape is necessary as wing size has an allometric component, and therefore has the ability to affect wing shape variation [56, 57]. Residuals are uncorrelated with the independent variables and are values of shape that do not contain the effect of size [58]. Finally, we performed a permutation test with 10,000 rounds on procrustes distance, as the dependent variable, and the residuals, as the independent variable to test if there is a significant difference between fore and hind wings of male and female *P*. *lydia* dragonfly.

## Results

### Body length, wing length, area, and wing loading

**Fig 2:**
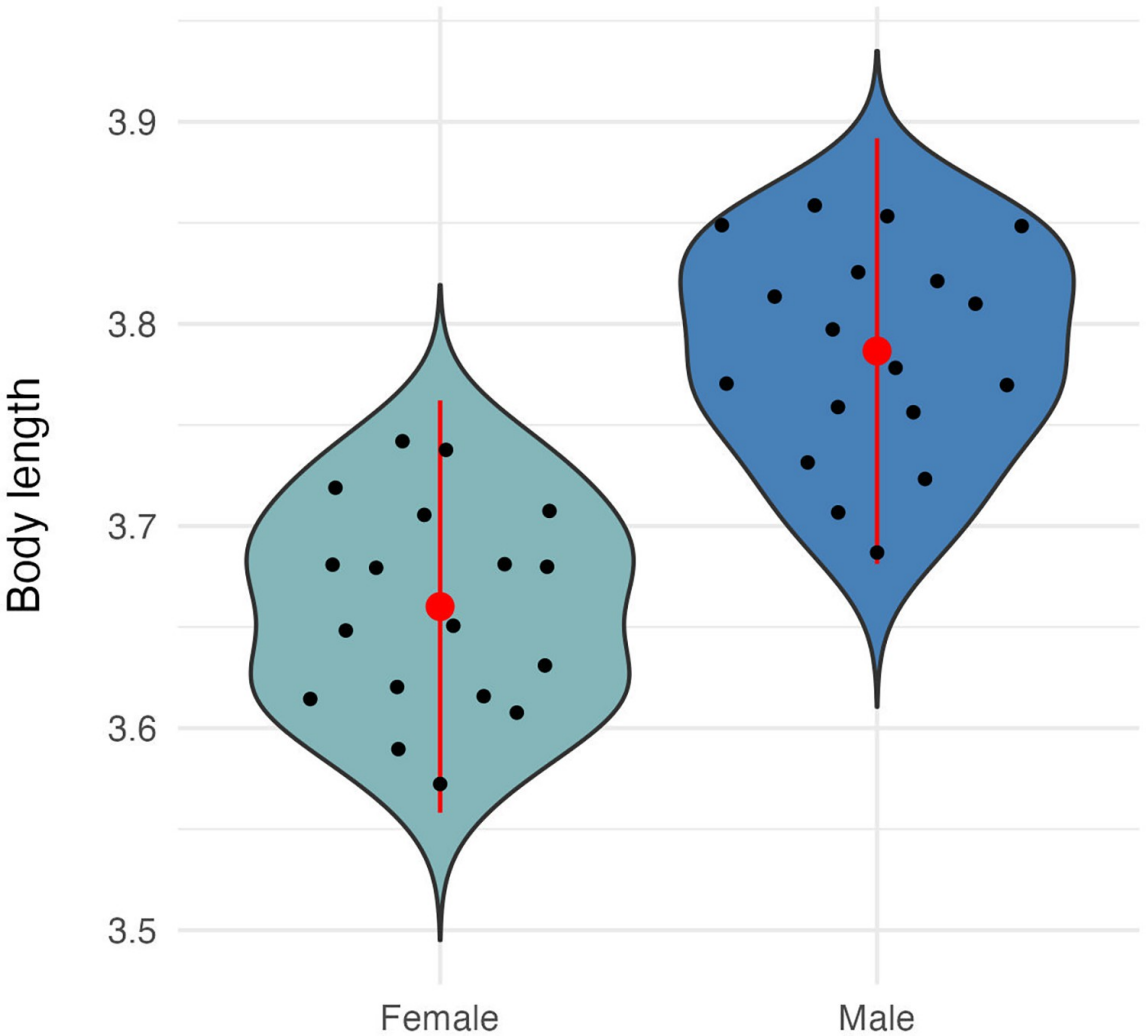
Mean body length for male and female common whitetail skimmer dragonfly (*Plathemis lydia*).

**Fig 3:**
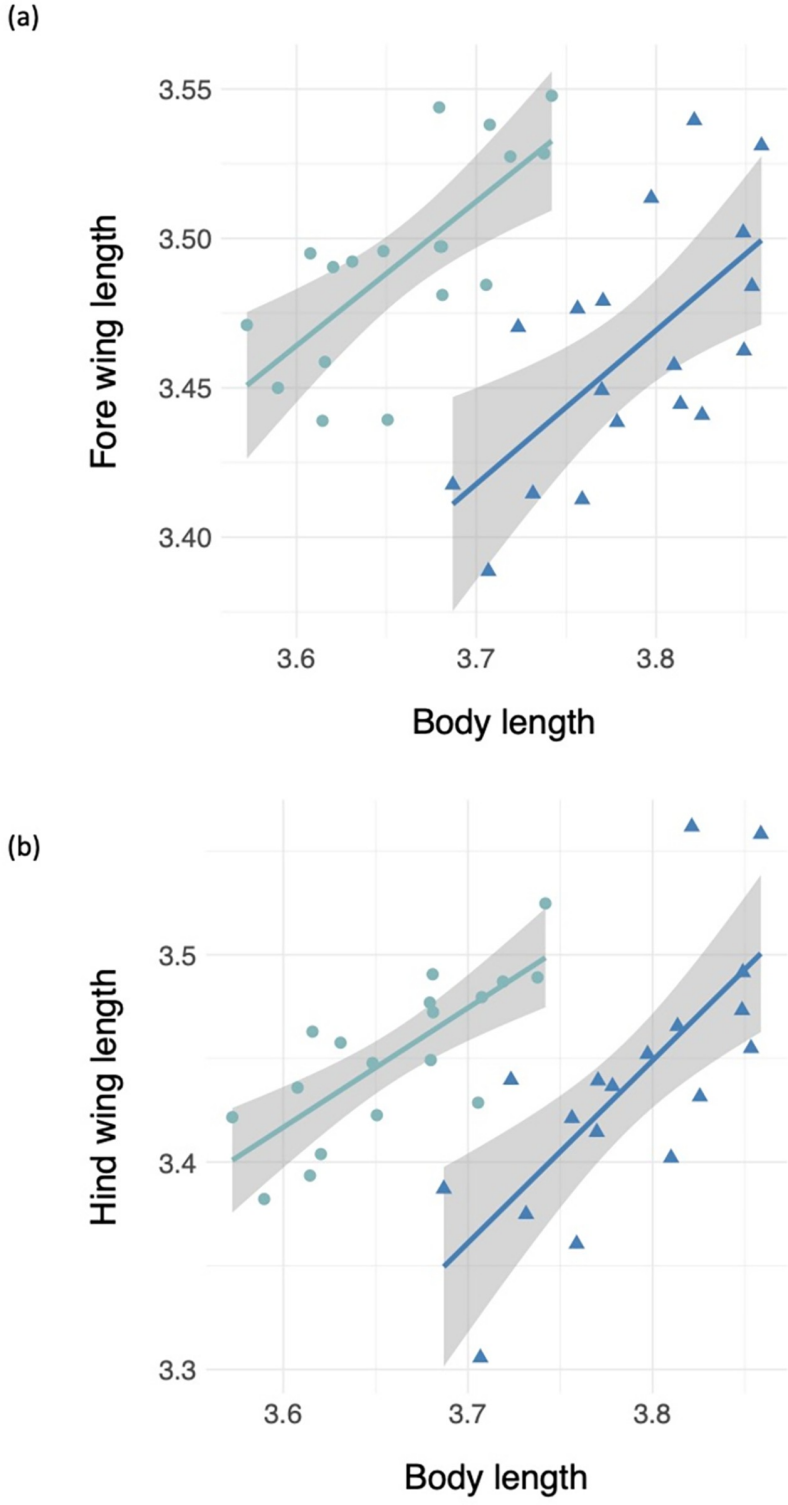
Wing length comparisons between male and female common whitetail skimmer dragonfly (*Plathemis lydia*): **(a)** Forewing length and **(b)** Hindwing length. The teal colored circles and slope represent data from female dragonflies whereas royal blue colored triangles and slope represent data from male *P*. *lydia* dragonflies.

**Fig 4:**
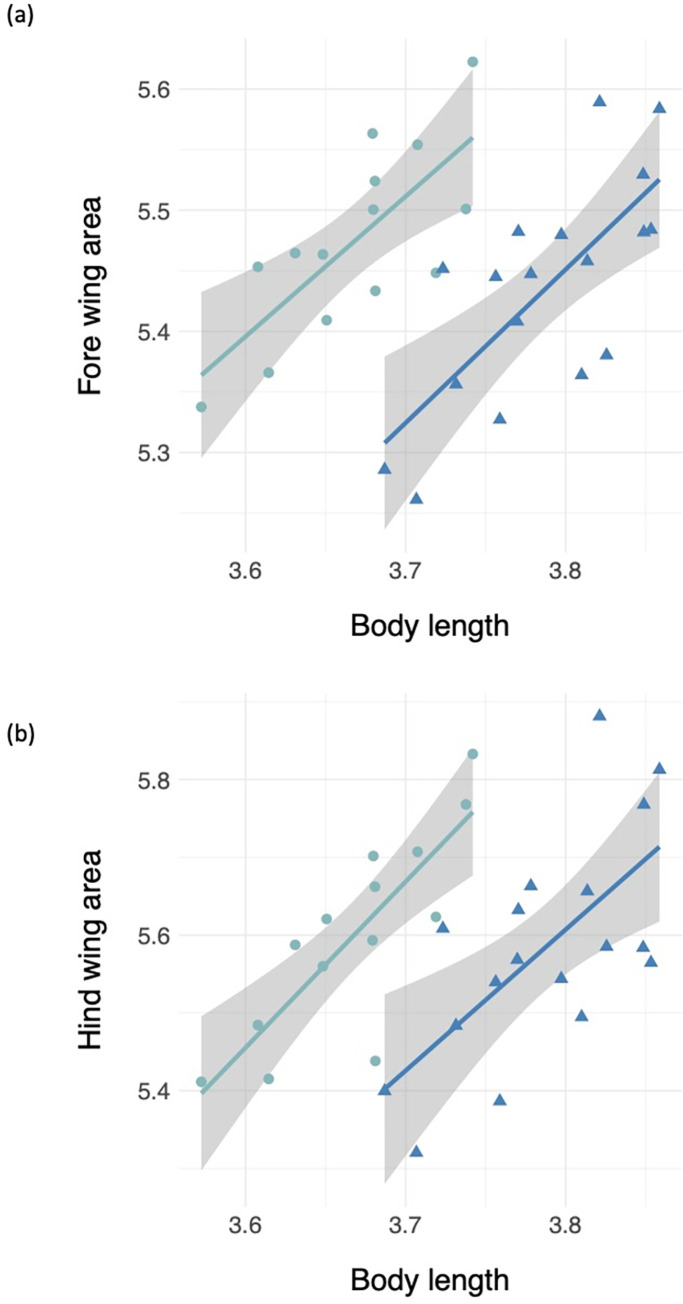
Wing area comparisons between male and female common whitetail skimmer dragonfly (*Plathemis lydia*): **(a)** Forewing area and **(b)** Hindwing area. The teal colored circles and slope represent data from female dragonflies whereas royal blue colored triangles and slope represent data from male *P*. *lydia* dragonflies.

**Fig 5:**
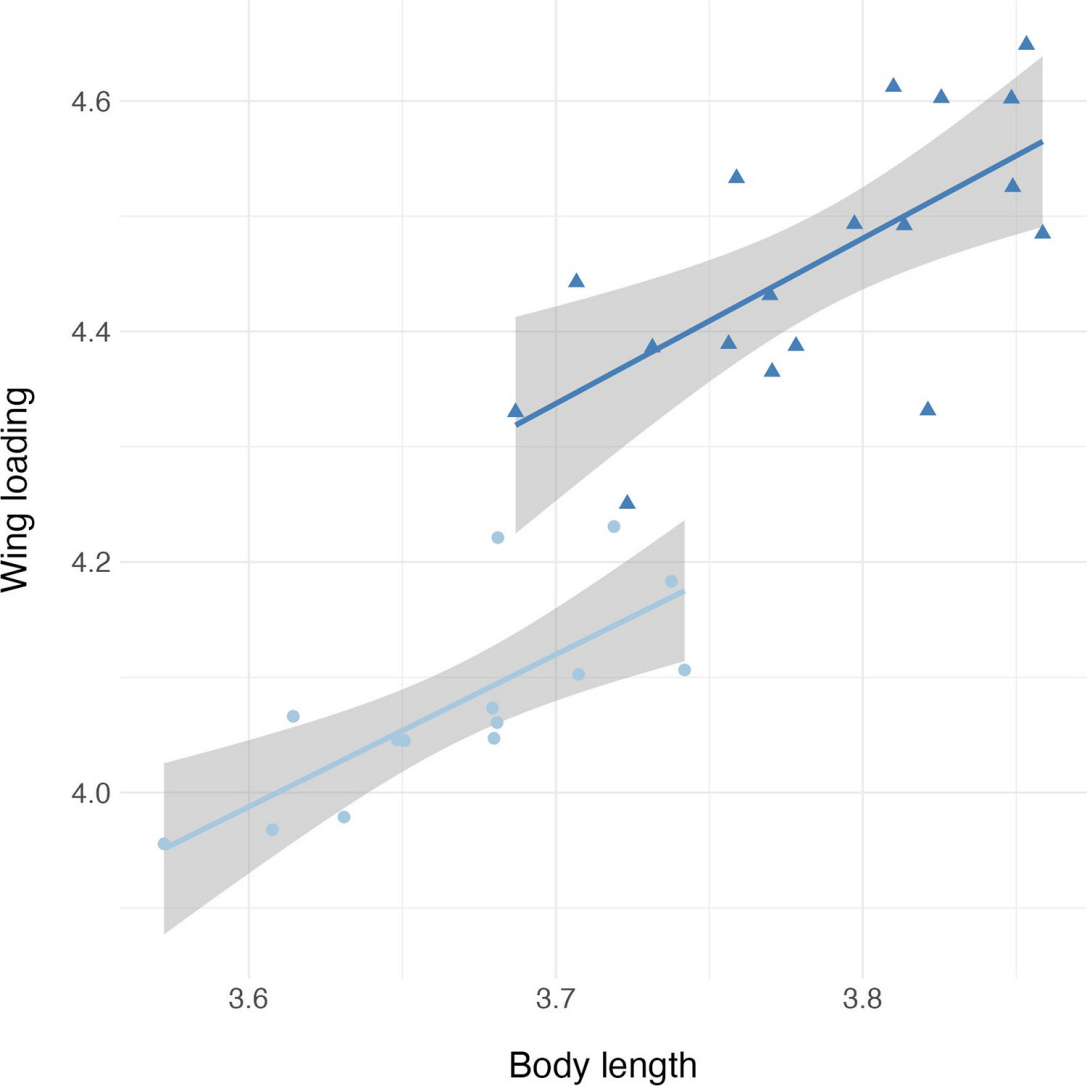
Wing loading comparisons between male and female common whitetail skimmer dragonfly (*Plathemis lydia*). The teal colored circles and slope represent data from female dragonflies whereas royal blue colored triangles and slope represent data from male *P*. *lydia* dragonflies.

Residuals for body length, fore and hind wing length, and fore and hind wing area did not deviate from normality (S1 Table). Average and standard deviations for all morphological measurements can be found in S2 Table. We found that male *Plathemis lydia* dragonflies were significantly longer than female dragonflies (t = -7.4424, df = 33.236, p-value = 1.427e-08) (Fig 2). When running an ANCOVA and using body length as a covariate, we found that male *P*. *lydia* dragonflies had significantly shorter fore and hind wings when compared to female dragonflies (Table 1 and Fig 3). In addition, we found that male *P*. *lydia* dragonflies had significantly lower fore and hind wing area when compared to female dragonflies (Table 1 and Fig 4). Furthermore, when comparing total wing loading between male and female *P*. *lydia* dragonflies, we found that male dragonflies had significantly higher wing loading than female dragonflies (Table 1 and Fig 5).

**Table 1:** Summary of morphological comparisons using an ANCOVA test.

Response variable	Effect	df	f	P-value
**Fore wing length**	Body length	1	0.5671	0.4569
	Sex	1	37.2196	8.11e-07
	Body length: Sex	1	0.0278	0.8687
	Residuals	32		
**Hind wing length**	Body length	1	8.5875	0.006199
	Sex	1	31.3211	3.501e-06
	Body length: Sex	1	1.6513	0.208002
	Residuals	32		
**Fore wing area**	Body length	1	5.1824	0.03066
	Sex	1	31.0303	5.848e-06
	Body length: Sex	1	0.0646	0.80118
	Residuals	28		
**Hind wing area**	Body length	1	10.521	0.003049
	Sex	1	21.122	8.368e-05
	Body length: Sex	1	0.205	0.654194
	Residuals	28		
**Wing loading**	Body length	1	205.4594	2.021e-14
	Sex	1	28.2541	1.171e-05
	Body length: Sex	1	0.0441	0.8353
	Residuals	28		

### Wing shape: Size variation and allometric effect

**Fig 6:**
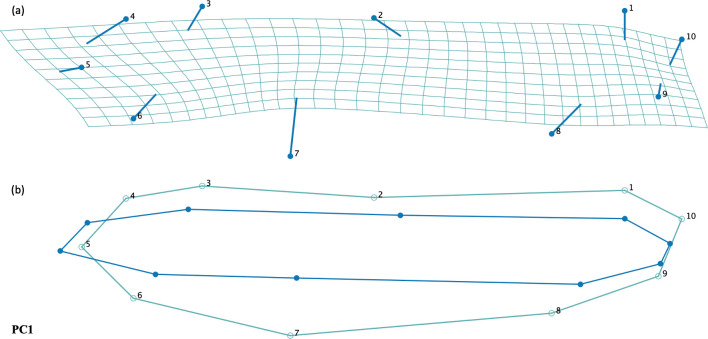
**(a)** Transformation grids and **(b)** warped outline drawings for fore wing shapes of male and female common whitetail skimmer dragonfly (*Plathemis lydia*).

**Fig 7:**
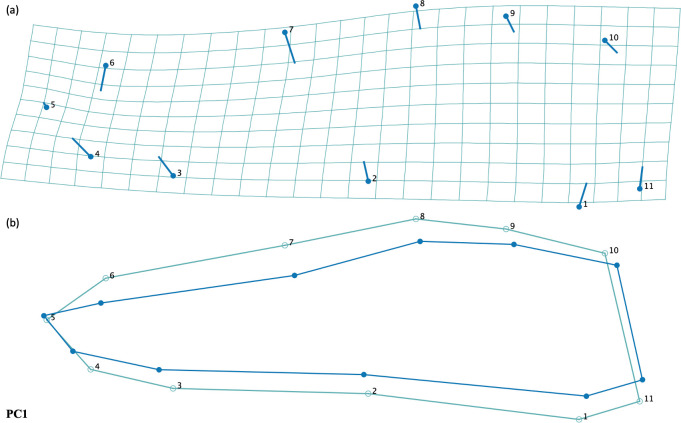
**(a)** Transformation grids and **(b)** warped outline drawings for hind wing shapes of male and female common whitetail skimmer dragonfly (*Plathemis lydia*).

We found that the fore wing centroid size between male and female *P*. *lydia* dragonflies was not significantly different (ANOVA: F = 2.18, df = 1, p-value = 0.1596) (Fig 6). In addition we did not find a significant difference in hind wing centroid size between male and female *P*. *lydia* dragonflies (ANOVA: F = 0.08, df = 1, p-value = 0.7762) (Fig 7). When controlling for size, we did not find a significant difference in fore wing shape between male and female dragonflies (Permutation test with 10,000 rounds, p-value = 0.0925) (Fig 6). However, when controlling for wing size, we did find a significant difference in hing wing shape between male and female *P*. *lydia* (Permutation test with 10,000 rounds, p -value = 0.0306). We found that male hind wings were narrower at the base and middle with pointier tips than females (Fig 7). For the fore wings, the first principal component accounted for 39.29% of the variance and the second principal component accounted for 25.89% of the variance (S3A Fig). For the hind wings, the first principal component accounted for 56.23% of the variance and the second principal component accounted for 16.70% of the variance (S3B Fig).

## Discussion

Our results show that male *Plathemis lydia* have a significantly longer body length, have significantly shorter fore and hind wings relative to body length, and higher wing loading when compared to females. We also found a significant difference in wing shape between male and female *P*. *lydia*, where the hind wings of males were narrower at the base and middle with pointier tips than the wings of females. Fore and hind wing centroid size between male and female *P*. *lydia* were not significantly different, showing similar trends to fore and hind wing area when not corrected for body length. *Plathemis lydia* is a sexually dichromatic organism, where males exhibit a conspicuous white abdomen and females exhibit a dark brown abdomen. The conspicuous abdomen of male *P*. *lydia* are not used during courtship with females but instead during male-male territorial chases [46, 47]. Therefore, due to enhanced visibility, it is likely that male *P*. *lydia* are detected more frequently by predators than females. Consequently, differences in selective pressures due to sexual dichromatism may have driven the disparities found in the fore and hind wing between male and female *P*. *lydia*.

*Plathemis lydia* exhibits male-biased sexual size dimorphism, where the body length of males are significantly longer than females. Sexual size dimorphism has been widely observed throughout animal taxa including birds [59], anurans [60], fish [61], and insects [62]. Across taxa, the evolution of larger body sizes has been found to be favored by both natural and sexual selection with larger body sizes being associated with higher fitness [63]. Larger body sizes may be sexually advantageous in predator escape [64], resource competition [65], and competition for mates [12].

Sexual selection through male-male competition strongly favors larger male body sizes, contributing to the evolution of male-biased sexual size dimorphism [66]. For instance, territorial bees have undergone selection for larger body sizes compared to females due to their highly territorial behavior and male-male competition as well as the resource defense polygyny mating system of the species [67]. Male-biased sexual size dimorphism has also been associated with an increase in male body size plasticity that is likely driven by sexual selection on males in insect species [68]. In the order Odonata, body size of territorial species is linked to measurements of male fitness such as longevity, mating rate, lifetime mating success, and territorial success [69, 70]. These measurements of fitness are potential drivers of male-biased sexual size dimorphism in *P*. *lydia*, especially considering the male-male competition over territorial resources in this species [71].

Male-male territorial chases are energetically costly and having wings that maximize abilities associated with territorial defense is essential [72]. Territorial chases in dragonflies often result in males being evicted from a claimed territory, hence, increased speed is a vital flight performance [47, 49]. Lohmann et al. [73] found, via simulation, that dragonflies with higher speed produce overshooting, which is an adaptive feature of male-male territorial chases. Overshooting is an aggressive interception strategy to pursue and effectively herd rival males from a territory while avoiding collision. Hence, morphological wing features that increase overall speed and energy efficiency should be selected for in dragonflies that perform male-male territorial chases.

Energetically efficient flight is dependent on wing shape, which is tightly linked with flight performance [40, 74]. For instance, wing shapes that reduce flight costs are long and narrow and associated with increased speed and agility [32–39]. Berwaerts et al. [40] found that long and narrow wings are correlated with high acceleration capacity. Male *P*. *lydia* have narrower hind wings compared to females suggesting they are adapted for high acceleration during territorial chases. However, *P*. *lydia* males have short fore and hind wings relative to body length. One explanation for shorter fore and hind wings is that the decrease in wing area allows for higher wing loading [41]. Wing loading, the ratio of weight to wing surface area of an organism, plays a significant role in flight speed and flight maneuverability [41, 42, 75]. Organisms with high wing loading have a smaller wing area relative to their mass which increases their flight speed whereas organisms with low wing loading have greater wing area relative to their mass which increases their flight maneuverability [41, 42]. It is likely that male *P*. *lydia* have an increase in energetically efficient flight due to the shape and size of their hind wings compared to females; which may be vital considering the male-male territorial chases that males perform. Additionally, in territorial odonate species, males with longer wing lengths have been found to defend territories for fewer days compared to males with shorter wings, suggesting that this may be another advantage of exhibiting shorter wings during male-male competition [76]. Increased agility and speed due to narrow wings and higher wing loading is likely to result in more effective exclusion of competitors from a dragonflies’ territory.

In addition to male-male territorial related stressors, male *P*. *lydia* are likely to experience predator related stressors due to enhanced visibility. Organisms with bright conspicuous coloration are more likely to be detected by predators than organisms with dull coloration, especially males in sexually dichromatic species [13–18]. Increased speed and agility are essential flying abilities for evading predation [48] and, similarly to male-male competition, morphological features that increase these flight performances should be selected for. Considering that male *P*. *lydia* exhibit enhanced visibility, it is likely that they are detected more often than females and therefore, more susceptible to predation. The morphological features that may have been adapted for territorial chases, narrow wings and high wing loading, may also be adapted for the speed and agility required for predator evasion.

Compared to male *P*. *lydia*, females have longer fore and hind wings relative to body length and lower wing loading. In addition, we found that females exhibited hind wings that were wider at the base and middle with blunt tips. Organisms that exhibit wide wings and lower wing loading are likely to have increased energy demand with reduced flight speed and increased flight maneuverability [32–39, 41]. Dakin et al. [77] found that species with low wing loading display turns that have faster rotations and are sharper. As a result, speed may not be an important aspect of flight performance for female *P*. *lydia* and instead high maneuverability has been selected for. Maneuverability in flight has two primary components, turning radius and speed, that may be subject to different selective pressures [75]. Components of maneuverability are impacted by the allocation of mass, specifically the center of mass for a species [75, 78]. Mass allocation could explain differences between male and female wing areas relative to body length. High maneuverability allows organisms to evade predation as it is associated with the ability to complete turns with small radii [42, 52]. Prey are generally smaller than their predators and this allows prey species to make turns that are quicker and sharper than predators, allowing them to escape predation despite being slow flyers [79]. In addition, studies suggest that the basal lobe of the hind wing is associated with gliding [37, 80] and therefore, it is likely that female *P*. *lydia* have increased gliding performance compared to males due to exhibiting wider hind wings.

## Conclusions

In conclusion, we investigated wing morphometric differences between sexes of a sexually dichromatic dragonfly, *P*. *lydia*). Male *P*. *lydia* display a conspicuous white abdomen whereas females display a dark brown abdomen. Due to differences in conspicuousness, it is likely that males are detected more often than females, and therefore, experience an increased level of predator related stressors. In addition, male *P*. *lydia* exhibit male-male territorial chases, which may cause increased sexual selection pressures. We found that male *P*. *lydia* had significantly shorter fore and hind wings relative to body length and higher wing loading when compared to females. In addition, we found that male *P*. *lydia* had hind wings that were narrower and pointier than females. The shape and size of male wings suggest that there have been adaptations associated with increased flight speed, agility, and acceleration capacity. Female *P*. *lydia* have wider hind wings that are likely adapted to increase flight maneuverability in the form of smaller turning radii. Considering that sexes of *P*. *lydia* dragonfly experience different selective pressures, the sexes have likely adapted different wing morphologies that offset these pressures.This is the first study to demonstrate wing morphological differences between the sexes in a sexually dichromatic dragonfly where males and females are undergoing different selective pressures due to variation in conspicuousness.

## Supporting information

S1 Table Summary of normally distributed residuals using a Shapiro-Wilk test. (DOCX)

S2 Table Average and standard deviation for all morphological features tested. (DOCX)

S1 Fig Measurements of fore and hind length (orange) and wing area (blue) of common whitetail skimmer dragonfly (*Plathemis lydia*). The dragonfly on the left is the male (a) and the dragonfly on the right is the female (b).
(DOCX)

S2 Fig Fore and hind wing landmarks used to capture wing length and wing shape of the common whitetail skimmer dragonfly (*Plathemis lydia*). Ten landmarks were used on the fore wing and eleven landmarks were used on the hind wing. The dragonfly on the left is the male (a) and the dragonfly on the right is the female (b).
(DOCX)

S3 Fig Principal components scores for **(a)** fore wing and **(b)** hind wing shape of the common whitetail skimmer dragonfly (*Plathemis lydia*). Teal colored circles points and mean confidence ellipse represent data from female *P*. *lydia* dragonflies whereas royal blue colored circles and mean confidence ellipse represent data from male *P*. *lydia* dragonflies.
(DOCX)
