# Supplementary material for: On the wings of dragons: Wing morphometric differences in the sexually dichromatic common whitetail skimmer dragonfly, Plathemis lydia (Odonata: Libellulidae)
Source: PLoS One. 2024 May 29;19(5):e0303690. doi: 10.1371/journal.pone.0303690 (PMC11135787; doi:10.1371/journal.pone.0303690)
Supplement: S1 Table — (DOCX) [file pone.0303690.s001.docx]

**S1 Table:** Summary of normally distributed residuals using a Shapiro-Wilk test.

| **Group** | **Sex** | **W** | **P-value** | **n** |
| --- | --- | --- | --- | --- |
| **Body length** | Male | 0.95341 | 0.481 | 18 |
|  | Female | 0.9585 | 0.5729 | 18 |
| **Fore wing length** | Male | 0.97345 | 0.8595 | 18 |
|  | Female | 0.94363 | 0.3339 | 18 |
| **Hind wing length** | Male | 0.93907 | 0.2794 | 18 |
|  | Female | 0.95642 | 0.5342 | 18 |
| **Fore wing area** | Male | 0.96204 | 0.6415 | 18 |
|  | Female | 0.9756 | 0.9412 | 14 |
| **Hind wing area** | Male | 0.94398 | 0.3385 | 18 |
|  | Female | 0.98344 | 0.9901 | 14 |
