# Supplementary material for: On the wings of dragons: Wing morphometric differences in the sexually dichromatic common whitetail skimmer dragonfly, Plathemis lydia (Odonata: Libellulidae)
Source: PLoS One. 2024 May 29;19(5):e0303690. doi: 10.1371/journal.pone.0303690 (PMC11135787; doi:10.1371/journal.pone.0303690)
Supplement: S2 Table — (DOCX) [file pone.0303690.s002.docx]

**S2 Table:** Average and standard deviation for all morphological features tested.

| **Group** | **Sex** | **Average** | **SD** |
| --- | --- | --- | --- |
| **Body length** | Male | 44.16 mm | 2.30 |
|  | Female | 38.91 mm | 1.98 |
| **Fore wing length** | Male | 31.91 mm | 1.33 |
|  | Female | 32.90 mm | 1.11 |
| **Hind wing length** | Male | 31.15 mm | 1.97 |
|  | Female | 31.56 mm | 1.19 |
| **Fore wing area** | Male | 230.00 mm^2^ | 20.99 |
|  | Female | 239.15 mm^2^ | 18.56 |
| **Hind wing area** | Male | 268.49 mm^2^ | 39.54 |
|  | Female | 272.68 mm^2^ | 35.54 |
| **Wing loading** | Male | 87.14 | 9.79 |
|  | Female | 59.20 | 5.20 |
