# Supplementary material for: On the wings of dragons: Wing morphometric differences in the sexually dichromatic common whitetail skimmer dragonfly, Plathemis lydia (Odonata: Libellulidae)
Source: PLoS One. 2024 May 29;19(5):e0303690. doi: 10.1371/journal.pone.0303690 (PMC11135787; doi:10.1371/journal.pone.0303690)
Supplement: S1 Fig — The dragonfly on the left is the male (a) and the dragonfly on the right is the female (b). (DOCX) [file pone.0303690.s003.docx]

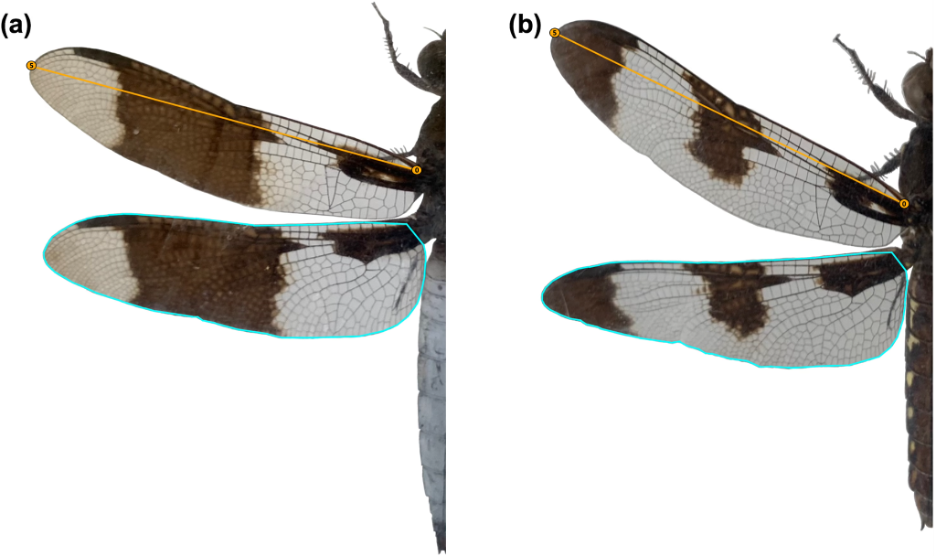


**S1 Fig:** Measurements of fore and hind length (orange) and wing area (blue) of common whitetail skimmer dragonfly (*Plathemis lydia*). The dragonfly on the **(a)** left is the male and the dragonfly on the **(b)** right is the female.
