# Supplementary material for: On the wings of dragons: Wing morphometric differences in the sexually dichromatic common whitetail skimmer dragonfly, Plathemis lydia (Odonata: Libellulidae)
Source: PLoS One. 2024 May 29;19(5):e0303690. doi: 10.1371/journal.pone.0303690 (PMC11135787; doi:10.1371/journal.pone.0303690)
Supplement: S2 Fig — Ten landmarks were used on the fore wing and eleven landmarks were used on the hind wing. The dragonfly on the left is the male (a) and the dragonfly on the right is the female (b). (DOCX) [file pone.0303690.s004.docx]

**
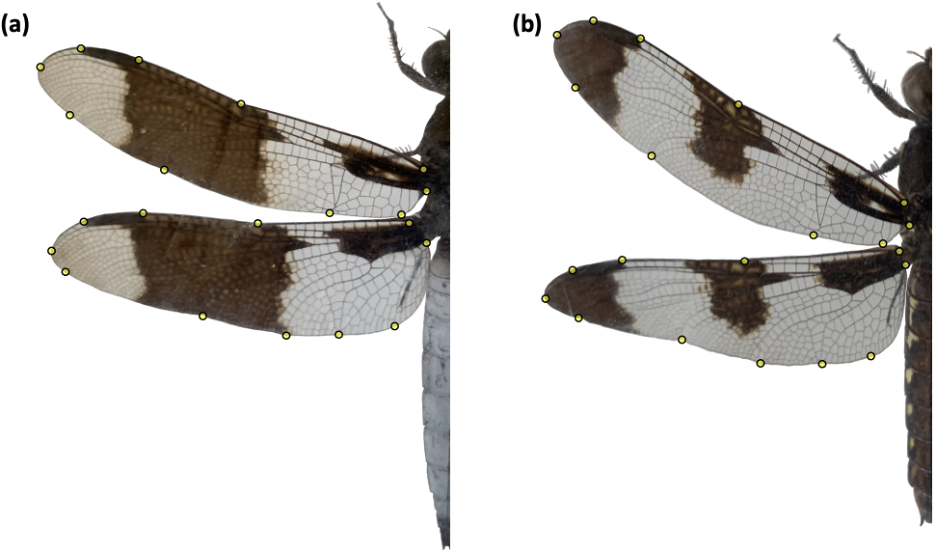
**

**S2 Fig:** Fore and hind wing landmarks used to capture wing length and wing shape of the common whitetail skimmer dragonfly (*Plathemis lydia*). Ten landmarks were used on the fore wing and eleven landmarks were used on the hind wing. The dragonfly on the **(a)** left is the male and the dragonfly on the **(b)** right is the female.
