# Supplementary material for: On the wings of dragons: Wing morphometric differences in the sexually dichromatic common whitetail skimmer dragonfly, Plathemis lydia (Odonata: Libellulidae)
Source: PLoS One. 2024 May 29;19(5):e0303690. doi: 10.1371/journal.pone.0303690 (PMC11135787; doi:10.1371/journal.pone.0303690)
Supplement: S3 Fig — Principal components scores for (a) fore wing and (b) hind wing shape of the common whitetail skimmer dragonfly (Plathemis lydia). Teal colored circles points and mean confidence ellipse represent data from female P. lydia dragonflies whereas royal blue colored circles and mean confidence ellipse represent data from male P. lydia dragonflies. (DOCX) [file pone.0303690.s005.docx]

**
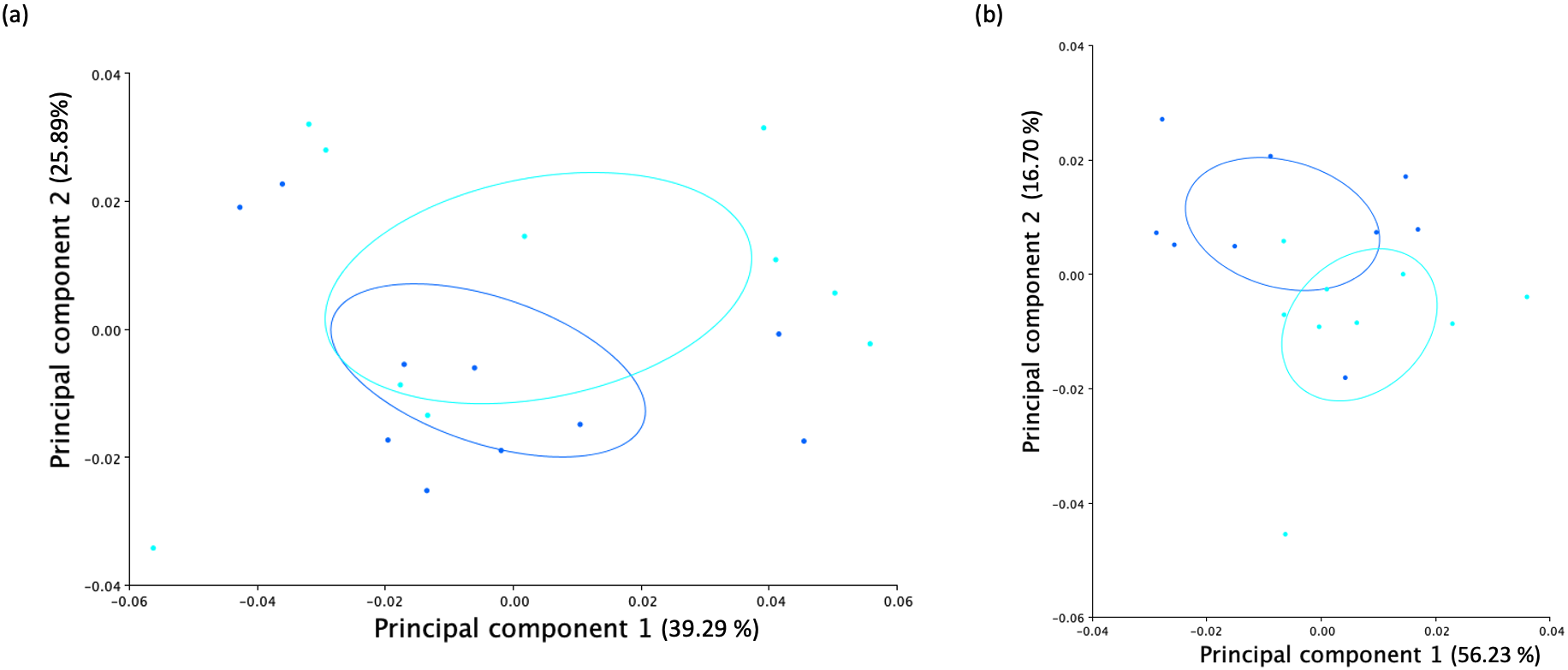
**

**S3 Fig:** Principal components scores for **(a)** fore wing and **(b)** hind wing shape of the common whitetail skimmer dragonfly (*Plathemis lydia*). Teal colored circles points and mean confidence ellipse represent data from female *P. lydia* dragonflies whereas royal blue colored circles and mean confidence ellipse represent data from male *P. lydia* dragonflies.
